# Combining the least cost path method with population genetic data and species distribution models to identify landscape connectivity during the late Quaternary in Himalayan hemlock

**DOI:** 10.1002/ece3.1840

**Published:** 2015-11-24

**Authors:** Haibin Yu, Yili Zhang, Linshan Liu, Wei Qi, Shicheng Li, Zhongjun Hu

**Affiliations:** ^1^ Key Laboratory of Land Surface Pattern and SimulationInstitute of Geographic Sciences and Natural Resource ResearchChinese Academy of Sciences (CAS)Beijing100101China; ^2^ School of Life SciencesSun Yat‐sen UniversityGuangzhou510275China; ^3^ CAS Center for Excellence in Tibetan Plateau Earth SciencesBeijing100101China; ^4^ University of Chinese Academy of SciencesBeijing100049China

**Keywords:** Gene flow, landscape genetics, least cost path method, phylogeography, Quaternary, species distribution models, Tibetan Plateau

## Abstract

Himalayan hemlock (*Tsuga dumosa*) experienced a recolonization event during the Quaternary period; however, the specific dispersal routes are remain unknown. Recently, the least cost path (LCP) calculation coupled with population genetic data and species distribution models has been applied to reveal the landscape connectivity. In this study, we utilized the categorical LCP method, combining species distribution of three periods (the last interglacial, the last glacial maximum, and the current period) and locality with shared chloroplast, mitochondrial, and nuclear haplotypes, to identify the possible dispersal routes of *T. dumosa* in the late Quaternary. Then, both a coalescent estimate of migration rates among regional groups and establishment of genetic divergence pattern were conducted. After those analyses, we found that the species generally migrated along the southern slope of Himalaya across time periods and genomic makers, and higher degree of dispersal was in the present and mtDNA haplotype. Furthermore, the direction of range shifts and strong level of gene flow also imply the existence of Himalayan dispersal path, and low area of genetic divergence pattern suggests that there are not any obvious barriers against the dispersal pathway. Above all, we inferred that a dispersal route along the Himalaya Mountains could exist, which is an important supplement for the evolutionary history of *T. dumosa*. Finally, we believed that this integrative genetic and geospatial method would bring new implications for the evolutionary process and conservation priority of species in the Tibetan Plateau.

## Introduction

The study of measurement and modeling of dispersal of organisms associated with genetic data is of growing interest in the field of landscape ecology (Tischendorf and Fahrig 2000; Sork and Smouse 2006; Wang et al. 2009; Lowe and Allendorf 2010). Evidence suggests that dispersal is a key processes determining the spatial population structure of species, in particular historical dispersal and resulting gene flow among populations affected by the Quaternary climate fluctuations, profoundly influenced the diversity of current species (e.g., Hewitt 2000, 2004; Lindborg and Eriksson 2004; Lessa et al. 2010; Sandel et al. 2011). Therefore, locating historical dispersal and assessing its effects are crucial in understanding the demographic and evolutionary history of species, and more practical, in providing significant illustrations for conserving endangered species in the face of climate change (Brown and Yoder 2015).

In past decades, the developing field of phylogeography has shown that a number of temperate plants experienced repeated expansion–contraction in species ranges during the late Quaternary period, and several hypothesized colonization routes from the glaciation refugia to ice‐free areas have been predicted on the European and North American continents (e.g., Taberlet et al. 1998; Soltis et al. 2006). For these studies, population demography associated with (re)colonization events can usually be inferred using coalescent theory and statistical methods (Nielsen and Wakeley 2001; Excoffier 2004; Hey and Nielsen 2004); however, the dispersal routes in a spatial context remain unclear. Fortunately, given habitat heterogeneity as a friction layer using geospatial and environmental data, several approaches such as the least cost path (LCP) method and circuit theory have made it possible to explore the location of dispersal corridors across the landscapes (Vignieri 2005; McRae and Beier 2007; Zeller et al. 2012; Graves et al. 2014). Among these methods, the LCP calculation coupled with species distribution models (SDMs) and population genetic data have been applied to identify landscape connectivity in a spatially explicit framework (Chan et al. 2011; Brown 2014). This integrated LCP method is mainly based upon two premises (Chan et al. 2011): one states that species distribution within suitable habitat could have facilitated population connectivity, namely high probability of occurrence in the SDMs has low cost to dispersal through the landscape matrix; the second precondition is that at molecular level, populations with shared haplotypes usually experienced dispersal between sample localities. Thus, if you obtained all the shared haplotypes between populations and past species distribution, it would be feasible to determine historical dispersal routes of plant species during the late Quaternary using the combined LCP method.

**Figure 1:**
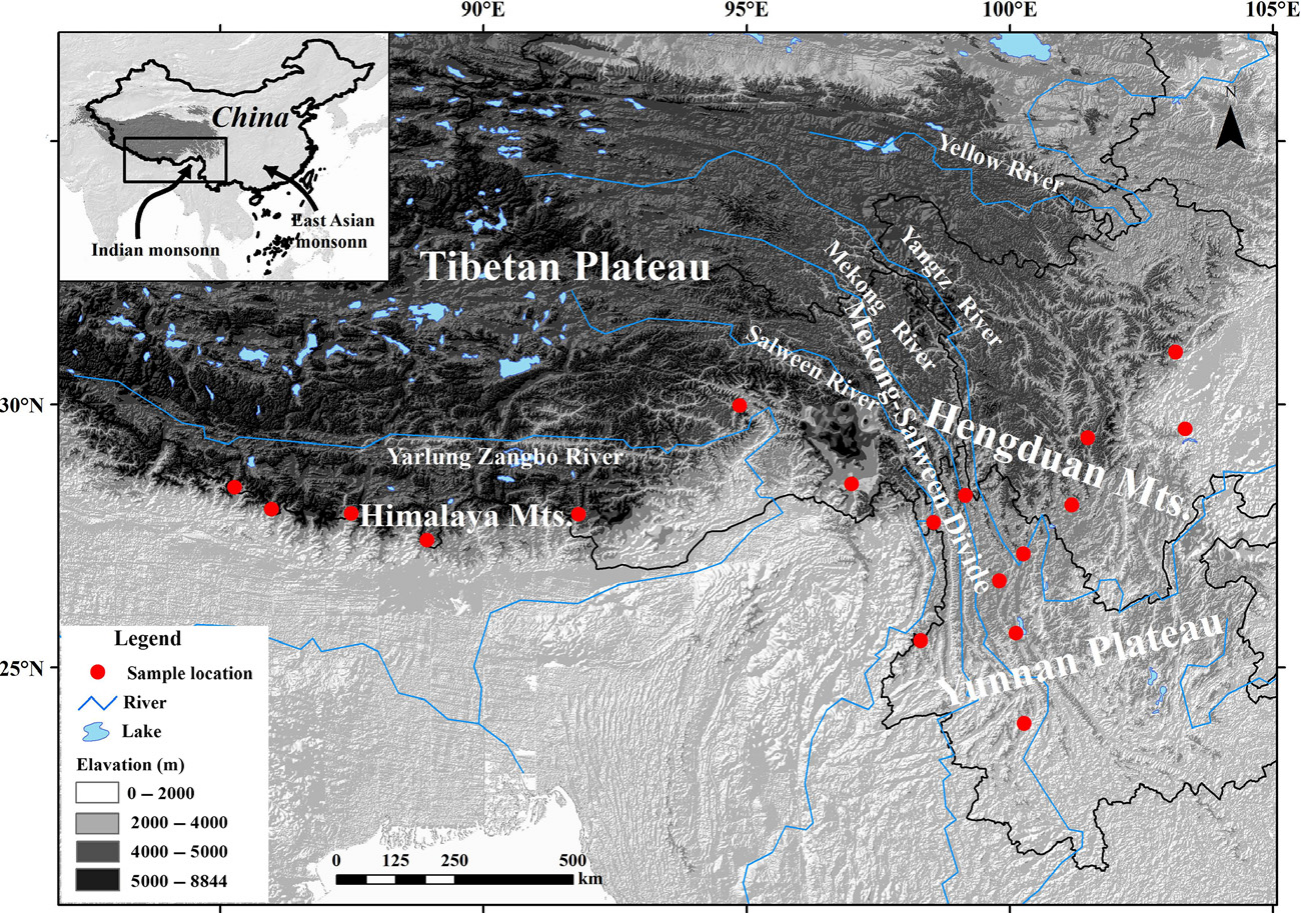
Localities of *Tsuga dumosa* and its surrounding environment.

Within the subalpine zone of the eastern and central Himalaya and the Hengduan Mountains (HHM) region, several cold‐inferring forest species that belong to *Picea*,* Abies*,* Tsuga,* and *Taxus*, are widely distributed (Singh and Singh 1987; Xu et al. 2014). During the late Quaternary, most of plants in the HHM region experienced little change in their distribution, resulting from stable climate and a large geographical barrier (“Mekong‐Salween Divide”) that hindered species dispersal in the Hengduan Mountains (Fig. 1; e.g., Opgenoorth et al. 2010; Li et al. 2011; Yue and Sun 2014). However, a few cold‐tolerant species, such as *Tsuga dumosa*,* Picea likiangensis,* and *Taxus wallichiana*, experienced unusual population expansion from the Hengduan Mountains to the Himalaya in this period (Cun and Wang 2010; Li et al. 2013; Liu et al. 2013); moreover, the population expansion mode of these cold‐tolerant species is quite contrary to the contraction reflection of species in other temperate regions when in response to the last glaciation (Taberlet et al. 1998; Soltis et al. 2006). Thus, a question should be raised, which whether or not several expansion routes could really exist in the HHM region during the late Quaternary, if they existed, we want to know the location of these dispersal corridors.

In this study, we chose a widespread subalpine species *T. dumosa* as our model to identify its dispersal routes. There are three ideal reasons for choosing this species: first of all, according to its previous phylogeographical work, a series of conclusions inferred from molecular evidence have revealed that this hemlock species underwent range expansion after the largest glaciation (0.8–0.6 Ma) in the HHM region (Cun and Wang 2010), which brings about a hypothesis that there could be one or several historical dispersal corridors exist in this region; secondly, because paternal chloroplast (cp), maternal mitochondrial (mt), and biparental nuclear (n) DNA adopted by its previous work represent various inheritance and dispersal features, if we use these three kinds of genomic markers, we can study the effects of different levels of gene flow through seed and pollen‐mediated; thus, a more comprehensive prediction of dispersal corridors become more convincing; eventually, substantial fossil records of *Tsuga* would provide actual evidence for the dispersal estimation. Therefore, for these three particular reasons, *T. dumosa* is a finer case study for exploring the dispersal corridors using the LCP method. For this study, our main objectives are (1) to identify the dispersal routes during the late Quaternary in the HHM region and assess their reliability from various evidence, and (2) to compare the concordance/discordance in dispersal corridors across time periods and molecular markers; (3) we expected to reveal some valuable implications for the evolutionary history and conservation priority of plants in the Tibetan Plateau (TP).

## Methods

### Determination of shared haplotypes

**Figure 2:**
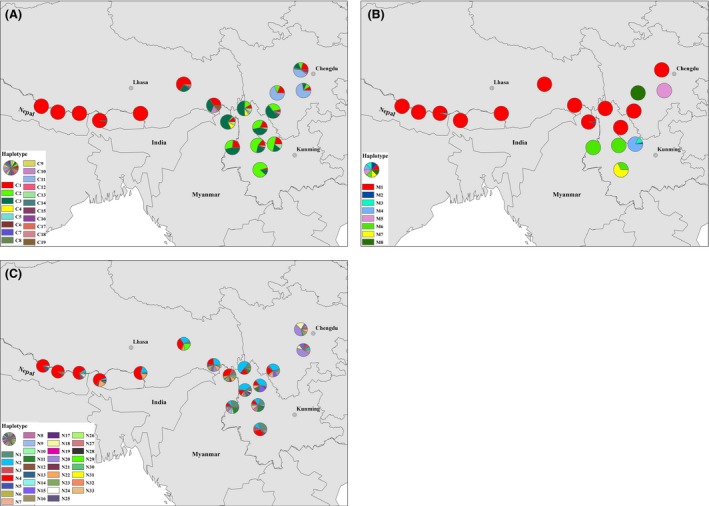
Haplotype distribution based on three genetic markers. (A) Chloroplast (cp) DNA; (B) Mitochondrial (mt) DNA; (C) Nuclear (n) DNA.

To explore the possible dispersal routes of *T. dumosa*, which populations have shared haplotypes needed to be firstly determined. According to the previous phylogeographical work from Cun and Wang (2010), they collected 18 sample sites (503 individuals), and 19, 8, and 90 haplotypes were determined for cpDNA, mtDNA, and nDNA, respectively. Among these haplotypes, 7, 4, and 33 are the number of shared haplotypes. For this study, we adopted the haplotype information of 18 localities, and populations with shared haplotypes from the three types of molecular markers were shown in the haplotype distribution maps (Fig. 2).

### Species distribution modeling

We used environmental and occurrence data to build species distribution models, identifying areas that *T. dumosa* could exist across three time periods. The three periods are the current conditions (~1950–2000), the last glacial maximum (LGM, ~21–18 ka) based on the Community Climate System Model (CCSM) and the last interglacial (LIG, ~140–120 ka). Environmental data in this study, including altitude and 19 bioclimate variables (Table S1), were obtained from the WorldClim database (http://www.worldclim.org/; Hijmans et al. 2005) with a resolution of 2.5 min for each layer. It is widely known that several climate variables are highly correlated with each other, in order to avoid overfitting of SDMs, we conducted an autocorrelation test of 19 bioclimate variables. Several bioclimate variables of the three periods that had relatively low Spearman's coefficients (*r *<* *0.75) were retained for subsequent analysis (Table S2).

For the second necessary data set for SDMs, species occurrence localities were mainly gathered from literature (Cun and Wang 2010), the Global Biodiversity Information Facility (GBIF, http://data.gbif.org), the Chinese Virtual Herbarium (CVH, www.cvh.org.cn) and field surveys in the year of 2013. For SDMs to be performed well, occurrence data require to be spatially independent, so the elimination of spatial clusters of localities is important for model calibration and evaluation. Because when spatial clusters of localities exist, models are often over‐fit toward environmental biases (reducing the model's ability to predict spatially independent data) and model performance values are inflated (Veloz 2009; Hijmans 2012; Boria et al. 2014). Hence, a spatially rarefy occurrence data method at several distances was applied according to topographic and climatic heterogeneity (Boria et al. 2014). Here, localities of occurrence were spatially filtered at 5, 15, and 25 km^2^ in areas of high, medium and low climatic heterogeneity, respectively. For the estimation of climatic heterogeneity, we conducted a principal component (PC) analysis for 19 bioclimate variables, and then we calculated the mean standard deviation of the first three climate PCs. Finally, the remaining 65 localities together with low correlated environmental layers were used for species distribution modeling.

In this study, we used the maximum entropy algorithm in MAXENT v3.3 (Phillips et al. 2006), to estimate current and past distributions of *T. dumosa*, with the settings for convergence threshold (10^−5^), number of iterations (500) and localities of occurrence divided into testing data sets and training data sets (20 and 80%, respectively). In addition, we carried out pairwise comparison of the binary SDMs for the three periods (e.g., from the last interglacial to the last glacial maximum) to predict range shifts since the last interglacial period.

### Visualizing dispersal corridors

**Figure 3:**
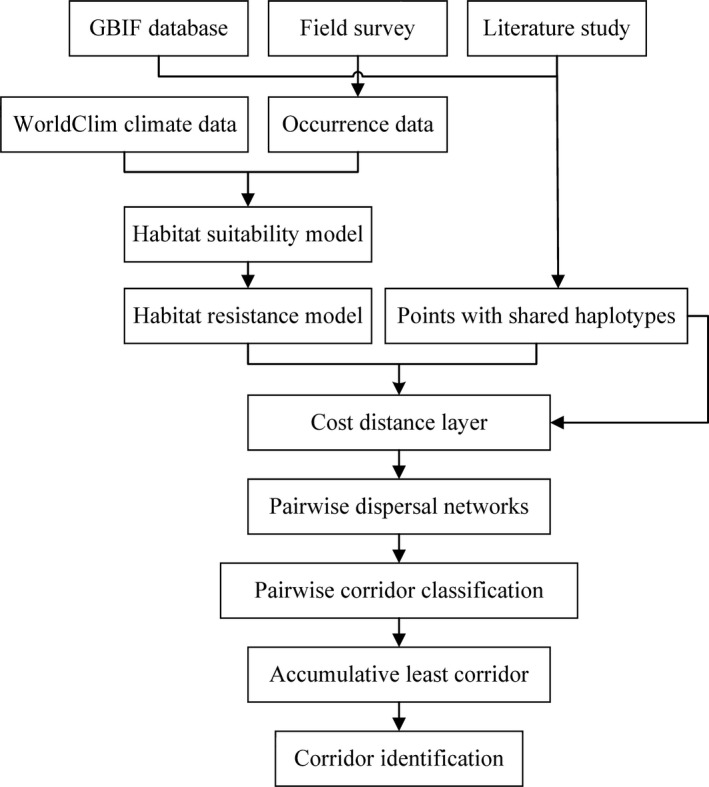
Research framework for this study.

By applying the LCP method, we mapped the dispersal routes of *T. dumosa* since the late Quaternary using SDMtoolbox (Brown 2014) in ArcGIS 10.1 (Environmental Systems Research Institute, Inc., Redlands, CA). The detailed procedures for this method were as follows (Fig. 3): firstly, we obtained a dispersal cost layer (resistance layer) by inverting the SDMs (1‐SDM), and subsequently applied the resistance layer to create a cost distance raster for each sample locality (18 localities in all); secondly, based on the cost distance raster, corridor layers were established between two localities that only had shared haplotypes; thirdly, while most previous studies (e.g., Vignieri 2005; Wang et al. 2009) always obtained a linear dispersal route, which may oversimplify the demographic process of species; hence, we applied the categorical LCP approach to better depict environmental heterogeneity in dispersal. Here, we classified the value of each corridor layer into four intervals (three cutoff values), if the lowest value of corridor layer was hypothesized as 1.00, the four intervals are 1.00–1.01, 1.01–1.02, 1.02–1.05, and 1.05–highest value, then these four intervals were reclassified as new values 5, 2, 1, and 0, respectively; finally, we summed up all of the pairwise reclassified corridor layers and standardized (0–1), and the dispersal maps of *T. dumosa* were eventually identified in an explicit landscape. For the dispersal corridors across three periods and molecular markers, the LCP approach was carried out nine times in all with same shared haplotypes patterns.

### Estimation of gene flow among regional groups

Estimation of gene flow among regional groups has been able to provide a fine proof for investigating dispersal corridors of species. As mentioned above, evidence suggests plants migrated from the Hengduan Mountains to the Himalaya, we hypothesized there was higher gene flow along this colonization route. To verify the hypotheses, according to the publication of Cun and Wang (2010), we divided the distribution range of *T. dumosa* into four regional groups: the Himalaya (Hi) group, the eastern Hengduan Mountains (EHe) group, the western and central Hengduan Mountains (W&CHe) group, and the southern Hengduan Mountains (SHe) group. To assess the level of gene flow among the four groups, a maximum‐likelihood estimation of migration rates based on coalescence theory was implemented using the software Migrate v3.6.4 (Beerli 1997; Beerli and Felsenstein 1999). Here, only adjacent groups were estimated based on cpDNA/mtDNA haplotype sequence (Accession No. HM162943‐HM162973, HM163057‐HM163059). Because in the study of Cun and Wang (2010), they did not provide the number of private haplotype in each population, that made it impossible for estimation of gene flow and subsequent analysis of mapping genetic divergence based on nDNA. For this approach, we concentrated on asymmetrical gene flow and unequal effective population sizes estimated from *F*_ST_ values. We conducted the program three times with different random seed numbers under the same condition (10 short chains, 500 trees used out of 50,000 sampled and 3 long chains, 5000 trees used out of 500,000 sampled).

### Mapping genetic divergence pattern

Geographical barriers usually lead to genetic divergence and hinder species dispersal. So a genetic divergence pattern could indicate which areas represent geographical barriers, and which areas with low values of genetic divergence could be suitable for dispersal. Here, we mapped the pattern of genetic divergence using the Genetic Landscape GIS Toolbox (Vandergast et al. 2011) in ArcGIS v10.1 (ESRI). Based on cpDNA/mtDNA haplotype sequence, the net number of sequence differences (*D*_A_; Nei and Li 1979) among 18 populations as the index of genetic divergence was firstly calculated using the ARLEQUIN v3.5 package (Excoffier et al. 2005) under the Tamura and Nei model of nucleotide evolution (Tamura and Nei 1993). Subsequently, genetic divergence values (*D*_A_) were mapped at the midpoints between populations and finally a continuous surface (namely genetic divergence pattern, 1‐km^2^ grid cell size) was created using an inverse distance weighted interpolation algorithm.

## Results

### Distribution changes since the last interglacial

**Figure 4:**
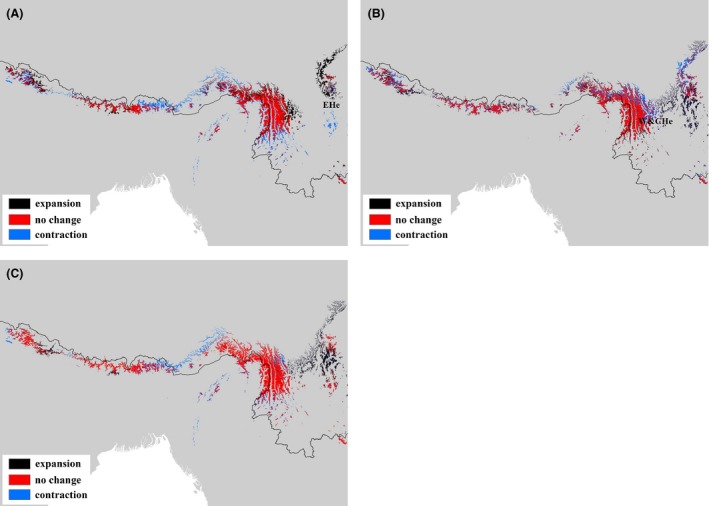
Distribution changes of *Tsuga dumosa* at three time intervals (A) from the last interglacial (LIG) to the last glacial maximum (LGM); (B) from the LGM to the current period; (C) from the LIG to the current period. Black, red, and blue colors represent range expansion, no change in range and range contraction, respectively. EHe represents eastern Hengduan Mountains, and W&CHe represents western and central Hengduan Mountains.

Due to cycles of glaciation in the Quaternary period, species distribution was commonly altered within the temperate regions. With no exception in this study, when comparing with species distribution of three periods, we identified the phased difference in ranges of *T. dumosa*. The detailed range changes showed that (1) from the last interglacial to the last glacial maximum, species distribution in the Himalaya mainly presented an east‐westward contraction and little downstream movement within the Himalayan valleys, in the meanwhile, for the EHe regional group, the species expanded northward (Fig. 4A); (2) after the last glacial maximum, the W&CHe group developed a wide range of eastward and northeastward expansion (Fig. 4B); (3) as a whole, the range shifts of Himalayan hemlock species have demonstrated little east‐westward and northeast‐southwestward trends since the last interglacial (Fig. 4C).

### Dispersal corridors across time periods and genetic markers

**Figure 5:**
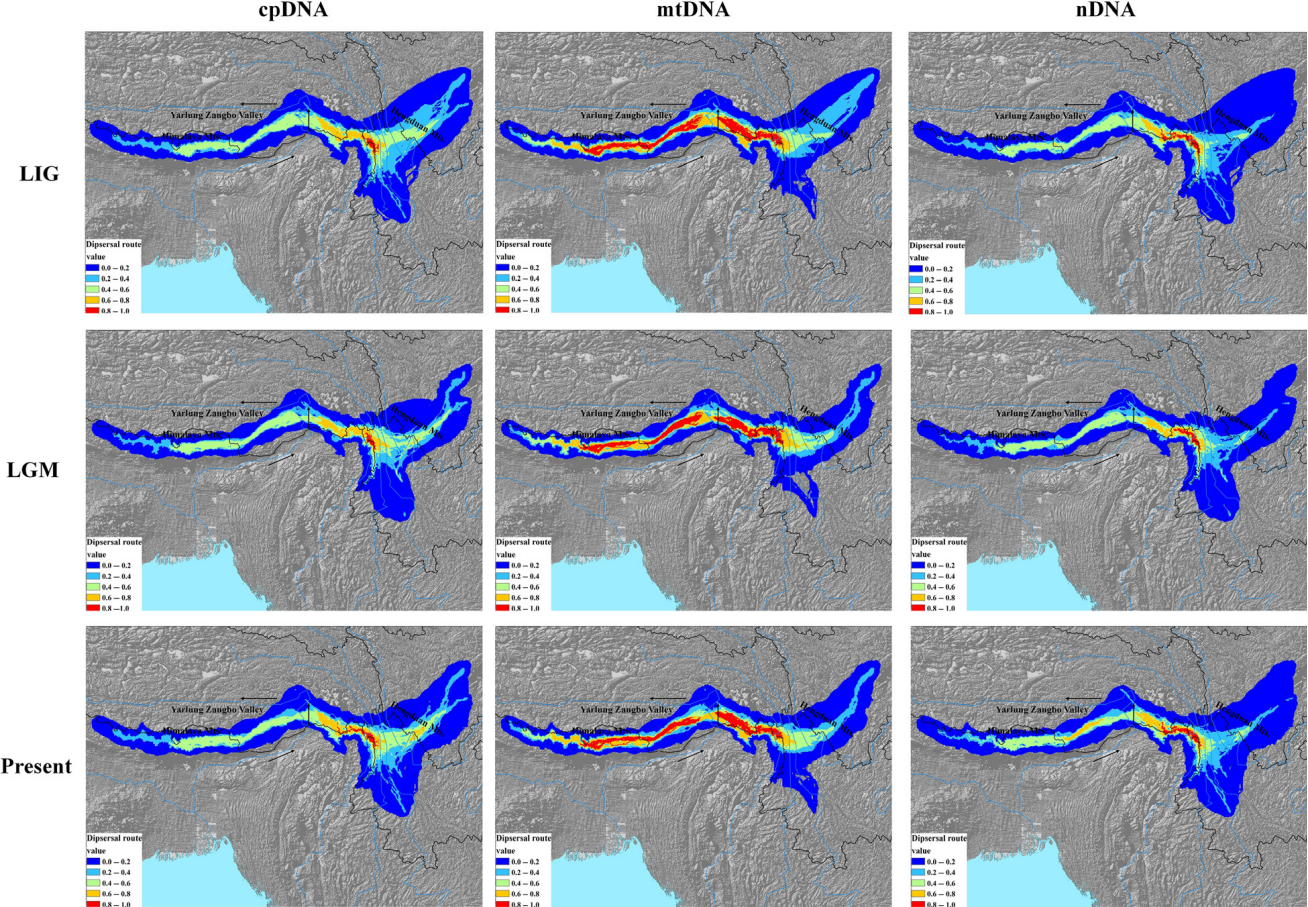
Potential dispersal routes of *Tsuga dumosa*. Data are originated from three periods (last interglacial (LIG), last glacial maximum (LGM), and present) and based on three genetic markers (chloroplast (cp) DNA, mitochondrial (mt) DNA, and nuclear (n) DNA). Arrows represent the direction of Indian monsoon.

Putative dispersal corridors in the three periods were visualized based on three genomic markers (Fig. 5). When comparing the dispersal routes across time periods and genetic markers, dispersal generally followed the southern slope of Himalaya, but the degree of likely dispersal varied across time and genomes, with higher dispersal in the present and in the mtDNA haplotypes. Another relatively higher area of dispersal from the western and central Hengduan Mountains to the eastern Hengduan Mountains was identified using cpDNA haplotypes. Besides, the dispersal route of *T. dumosa* is partially overlaid with the Yarlung Zangbo valley; we assumed this valley might be another important historical dispersal corridor.

### Patterns of gene flow and genetic divergence

**Figure 6:**
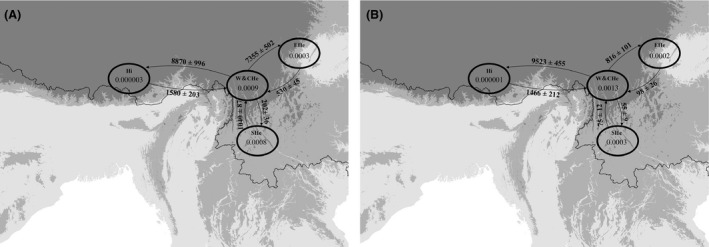
Estimation of gene flow among four regional groups based on cpDNA/mtDNA haplotype sequence (A) chloroplast (cp) DNA; (B) mitochondrial (mt) DNA. Arrows represent the direction of gene flow; numbers around the arrow represent mutation‐scaled immigration rates (M) and numbers in the circle represent mutation‐scaled the effective population size (Θ).

**Figure 7:**
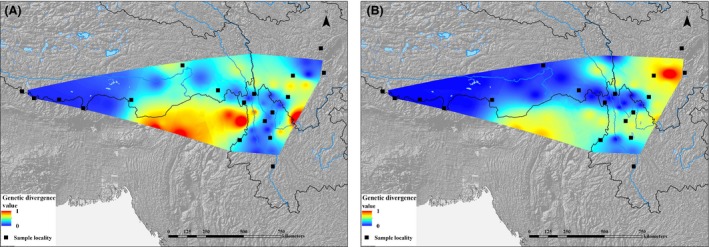
Pattern of genetic divergence among populations (A) chloroplast (cp) DNA; (B) mitochondrial (mt) DNA. Areas with low genetic divergence value are as a reflection of strong gene flow among populations.

According to gene flow patterns estimated from coalescent analyses based on cp/mt DNA (Fig. 6), the path from the Hengduan Mountains to the Himalaya had strong gene flow, which was mostly overlapped with the Himalayan dispersal corridor. Further, within the genetic divergence map (Fig. 7), area of Himalaya had low genetic divergence value, suggesting that no obvious barrier existed in this dispersal area during the Quaternary.

## Discussion

### Himalayan dispersal route and other dispersal corridors in the TP

By virtue of the LCP approach (which is commonly used in the field of landscape ecology), integrated with SDMs and molecular data, we have been able to identify the dispersal corridors of *T. dumosa* in the late Quaternary. In the TP, one directional dispersal approximately from the Hengduan Mountains to the Himalaya has been repeatedly assumed by several phylogeographical studies (e.g., Yang et al. 2008; Li et al. 2013; Liu et al. 2013), until now, we understand the location of the dispersal route for this coniferous forest species, which might have migrated along the southern slope of the Himalaya from the Hengduan Mountains during the late Quaternary period. Meanwhile, series of consequences in this study, such as distribution changes since the last interglacial, the high strength of gene flow and no noticeable barriers in this direction, also imply the existence of this route. Moreover, a wide occurrence of *Tsuga* pollen records (Cun and Wang 2010) on the southern slope of the Himalaya provides more convincingly evidence.

Apart from the Himalayan route, we assumed the Yarlung Zangbo Valley may be another dispersal corridor in the HHM region. According to a small amount of Quaternary *Tsuga* pollen and pollen of other coniferous species found within the valley (Song and Liu 1982; Kong et al. 1996; Tang and Shen 1996), it suggests that coniferous forests ever distributed in this valley. In the other hand, we know that the Yarlung Zangbo Valley with an average altitude below 4000 m is the largest vapor channel in the TP (Lin and Wu 1990), the features of lower topography together with strong airflow were likely to be helpful in the movement of seed and pollen of species. As a result, all above‐mentioned evidence demonstrates that the Yarlung Zangbo Valley may be another historical dispersal path. Furthermore, with regard to the sympatric alpine species *Pedicularis longiflora*, the LCP estimation for its dispersal corridor also focused on the area of Yarlung Zangbo Valley (Yu et al. 2014).

### Concordance and discordance in dispersal corridors across time periods and genetic markers

Generally, common geological history and climate conditions may lead to similar biogeographic patterns (Taberlet et al. 1998; Soltis et al. 2006). In view of the LCP method, the similar patterns containing species distributions across time periods and spatial haplotype structures among different genetic markers could lead to congruence in dispersal routes. For the hemlock species within the HHM, one side, the relatively stable climate during the late Quaternary region usually resulted in little change of species distribution (Cun and Wang 2010; Li et al. 2013; Liu et al. 2013), generating the almost consistent in dispersal areas across time. From another point of view, the Indian monsoon offered strong driving force to make wind‐dispersed pollen and seed move from the Hengduan Mountain to the Himalaya, accounting for the purification of haplotypes because of successive founder effects, and finally leading to similar haplotype patterns of three genetic markers. Consequently, although cpDNA, mtDNA, and nDNA reflect propagation characteristics of pollen and seed, the relative concordance in dispersal corridors was still exhibited across the three genomic markers.

Even if the overall accordance in dispersal routes was apparent, little distinction was still detected, especially for the degree of dispersal identified by different genomes. In hemlock species, the inheritance of mtDNA is typical maternal, reflecting seed movement exclusively (Neale and Sederoff 1989). Because seed dispersal was limited, it is more liable to generate genetic differentiation and haplotypes purification among the Himalayan populations. Go back to the LCP method, haplotype composition of colonized populations is more closer, indicating the higher degree of dispersal along the colonization route; in contrast, the paternal cpDNA and biparental nDNA, both represent the feature of pollen dispersal (nDNA also represents seed dispersal). Although extensive pollen transmissions occurred among populations, it would counteract the dispersal strengthen in colonization direction, resulting in lower level of dispersal than mtDNA along the Himalaya Mountains. Additionally, because tracing the imprint of historical seed colonization could reveal the true demographic history of species (Schaal et al. 1998), along with our results, thus we assumed that the estimation of dispersal route based on maternal inheritance of genome may be more reliable.

### Implications for the evolutionary history and conservation of alpine plants

Undoubtedly, determining the location of dispersal is an important supplement for the evolutionary history of species. The dispersal routes we identified would bring valuable insights to other alpine species in the HHM region. Except for *T. dumosa*, numerous alpine species migrate from the Hengduan Mountains to the Himalaya during the late Tertiary or Quaternary, we speculated they may spread along the southern slope of Himalaya or Yarlung Zangbo Valley or other uncovered corridors. Besides the location of dispersal routes, we really want to understand what factors drive the dispersal within the HHM region? It is well known that the Hengduan Mountains was regarded as an important region of origin and radiation of alpine plants and refugia during the Quaternary glaciation (Wu 1988a,b; Qiu et al. 2011; Yu and Zhang 2013). When they were confronted with rapid uplift of the TP in the late Tertiary and cycles of climatic oscillations in the Quaternary, in order to respond to the vast environmental changes, various evolutionary processes took place, such as local adaptation, long distance dispersal, and recolonization into the interior of the TP from glacial refugia after the glaciation. Besides, the Indian monsoon and East Asian monsoon originated in the late Miocene (An et al. 2001) also could offer continuous force to facilitate plant pollen and seed spread westward to the TP interior. Thus, during the Quaternary period, both Quaternary climatic fluctuations and Indian monsoon contributed to promote the dispersal of alpine plants in the HHM region (Liu et al. 2014; Wen et al. 2014).

The higher degree of dispersal area we identified is equally important for the conservation of species diversity in the HHM region. Based on the trends of species range shift responding to past climate change, we predict plant species will experience expansion toward the Himalaya Mountains under the future global warming. Thus, the Himalaya dispersal area should be prioritized to protect when making conservation strategies. Besides, in face of ongoing habitat destruction relating to human activities, such as building hydropower station and increasing number of tourists, weighed and strategic management for dispersal corridor should be taken into account.

## Conclusion

Our main objective in this study was to identify the dispersal routes of *T. dumosa*. Superficially, this appeared to be relatively straight‐forward, however, we realized that it could complement the evolutionary history of alpine plants in the TP. We can imagine that during the Quaternary period, in response to cycles of glaciation, plant species may have migrated along the Himalaya Mountains from the Hengduan Mountains, and evidence now demonstrates that this Himalayan dispersal route actually existed. However, we suggest that this may not be the only path from the Hengduan Mountains to the interior of the TP, there might be several corridors to be identified till more species involve to predict and more fossil records were discovered in future studies. Furthermore, for some endangered species, the estimation of potential dispersal activity is important for their conservation under future climate change.

## Conflict of Interest

None declared.

## Supporting information


**Table S1.** Codes used for 19 bioclimatic variables.
**Table S2.** Spearman's rank correlation coefficient (*r*) for the non‐eliminated bio‐climate predictors in the three periods.

## References

[ece31840-bib-0001] An, Z. S. , J. E. Kutzbach , W. L. Prell , and S. C. Porter . 2001 Evolution of Asian monsoons and phased uplift of the Himalaya‐Tibetan Plateau since Late Miocene times. Nature 411:62–66. 11333976 10.1038/35075035

[ece31840-bib-0002] Beerli, P. 1997 Migrate 0.7: documentation and program, part of LAMARC. Available at: http://evolution.genetics.washington.

[ece31840-bib-0003] Beerli, P. , and J. Felsenstein . 1999 Maximum‐likelihood estimation of migration rates and effective population numbers in two populations using a coalescent approach. Genetics 152:763–773. 10353916 10.1093/genetics/152.2.763 PMC1460627

[ece31840-bib-0004] Boria, R. A. , L. E. Olson , S. M. Goodman , and R. P. Anderson . 2014 Spatial filtering to reduce sampling bias can improve the performance of ecological niche models. Ecol. Modell. 275:73–77.

[ece31840-bib-0005] Brown, J. L. 2014 SDMtoolbox: a python‐based GIS toolkit for landscape genetic, biogeographic and species distribution model analyses. Methods Ecol. Evol. 5:694–700. 10.7717/peerj.4095 PMC5721907 29230356

[ece31840-bib-0006] Brown, J. L. , and A. D. Yoder . 2015 Shifting ranges and conservation challenges for lemurs in the face of climate change. Ecol. Evol. 5:1131–1142. 25859320 10.1002/ece3.1418 PMC4377258

[ece31840-bib-0007] Chan, L. M. , J. L. Brown , and A. D. Yoder . 2011 Integrating statistical genetic and geospatial methods brings new power to phylogeography. Mol. Phylogenet. Evol. 59:523–537. 21352934 10.1016/j.ympev.2011.01.020

[ece31840-bib-0008] Cun, Y. , and X. Wang . 2010 Plant recolonization in the Himalaya from the southeastern Qinghai‐Tibetan Plateau: geographical isolation contributed to high population differentiation. Mol. Phylogenet. Evol. 56:972–982. 20472080 10.1016/j.ympev.2010.05.007

[ece31840-bib-0009] Excoffier, L. 2004 Patterns of DNA sequence diversity and genetic structure after a range expansion: lessons from the infinite‐island model. Mol. Ecol. 13:853–864. 15012760 10.1046/j.1365-294x.2003.02004.x

[ece31840-bib-0010] Excoffier, L. , G. Laval , and S. Schneider . 2005 Arlequin (version 3.0): an integrated software package for population genetics data analysis. Evol. Bioinform. Online 1:47. 19325852 PMC2658868

[ece31840-bib-0011] Graves, T. , R. B. Chandler , J. A. Royle , P. Beier , and K. C. Kendall . 2014 Estimating landscape resistance to dispersal. Landsc. Ecol. 29:1201–1211.

[ece31840-bib-0012] Hewitt, G. 2000 The genetic legacy of the Quaternary ice ages. Nature 405:907–913. 10879524 10.1038/35016000

[ece31840-bib-0013] Hewitt, G. M. 2004 Genetic consequences of climatic oscillations in the Quaternary. Philos. Trans. R. Soc. Lond. B Biol. Sci. 359:183–195. 15101575 10.1098/rstb.2003.1388 PMC1693318

[ece31840-bib-0014] Hey, J. , and R. Nielsen . 2004 Multilocus methods for estimating population sizes, migration rates and divergence time, with applications to the divergence of *Drosophila pseudoobscura* and *D. Persimilis* . Genetics 167:747–760. 15238526 10.1534/genetics.103.024182 PMC1470901

[ece31840-bib-0015] Hijmans, R. J. 2012 Cross‐validation of species distribution models: removing spatial sorting bias and calibration with a null model. Ecology 93:679–688. 22624221 10.1890/11-0826.1

[ece31840-bib-0016] Hijmans, R. J. , S. E. Cameron , J. L. Parra , P. G. Jones , and A. Jarvis . 2005 Very high resolution interpolated climate surfaces for global land areas. Int. J. Climatol. 25:1965–1978.

[ece31840-bib-0017] Kong, Z. C. , N. Q. Du , and S. F. Shan . 1996 A preliminary study of vegetational changes in space‐time on Qinghai‐Xizang Plateau since Late Cenozoic. Acta Micropalaeontol. Sin. 19:339–351.

[ece31840-bib-0018] Lessa, E. P. , G. D. Elia , and U. F. Pardinas . 2010 Genetic footprints of late Quaternary climate change in the diversity of Patagonian‐Fueguian rodents. Mol. Ecol. 19:3031–3037. 20618900 10.1111/j.1365-294X.2010.04734.x

[ece31840-bib-0019] Li, Y. , S. Zhai , Y. Qiu , Y. Guo , X. Ge , and H. P. Comes . 2011 Glacial survival east and west of the ‘Mekong‐Salween Divide’ in the Himalaya‐Hengduan mountains region as revealed by AFLPs and cpDNA sequence variation in *Sinopodophyllum hexandrum* (Berberidaceae). Mol. Phylogenet. Evol. 59:412–424. 21296173 10.1016/j.ympev.2011.01.009

[ece31840-bib-0020] Li, L. , R. J. Abbott , B. Liu , Y. Sun , L. Li , J. Zou , et al. 2013 Pliocene intraspecific divergence and Plio‐Pleistocene range expansions within *Picea likiangensis* (Lijiang spruce), a dominant forest tree of the Qinghai‐Tibet Plateau. Mol. Ecol. 22:5237–5255. 24118118 10.1111/mec.12466

[ece31840-bib-0021] Lin, Z. Y. , and X. D. Wu . 1990 A preliminary analysis about the tracks of moisture transportation on the Qinghai‐Xizang Plateau. Geogr. Res. 9:33–40.

[ece31840-bib-0022] Lindborg, R. , and O. Eriksson . 2004 Historical landscape connectivity affects present plant species diversity. Ecology 85:1840–1845.

[ece31840-bib-0023] Liu, J. , M. Möller , J. Provan , L. M. Gao , R. C. Poudel , and D. Z. Li . 2013 Geological and ecological factors drive cryptic speciation of yews in a biodiversity hotspot. New Phytol. 199:1093–1108. 23718262 10.1111/nph.12336

[ece31840-bib-0024] Liu, J. Q. , Y. W. Duan , G. Hao , X. J. Ge , and H. Sun . 2014 Evolutionary history and underlying adaptation of alpine plants on the Qinghai‐Tibet Plateau. J. Syst. Evol. 52:241–249.

[ece31840-bib-0025] Lowe, W. H. , and F. W. Allendorf . 2010 What can genetics tell us about population connectivity? Mol. Ecol. 19:3038–3051. 20618697 10.1111/j.1365-294X.2010.04688.x

[ece31840-bib-0026] McRae, B. H. , and P. Beier . 2007 Circuit theory predicts gene flow in plant and animal populations. P. Natl Acad. Sci. USA 104:19885–19890. 10.1073/pnas.0706568104 PMC2148392 18056641

[ece31840-bib-0027] Neale, D. B. , and R. R. Sederoff . 1989 Paternal inheritance of chloroplast DNA and maternal inheritance of mitochondrial DNA in loblolly pine. Theor. Appl. Genet. 77:212–216. 24232531 10.1007/BF00266189

[ece31840-bib-0028] Nei, M. , and W. Li . 1979 Mathematical model for studying genetic variation in terms of restriction endonucleases. Proc. Natl Acad. Sci. USA 76:5269–5273. 291943 10.1073/pnas.76.10.5269 PMC413122

[ece31840-bib-0029] Nielsen, R. , and J. Wakeley . 2001 Distinguishing migration from isolation: a Markov chain Monte Carlo approach. Genetics 158:885–896. 11404349 10.1093/genetics/158.2.885 PMC1461674

[ece31840-bib-0030] Opgenoorth, L. , G. G. Vendramin , K. Mao , G. Miehe , S. Miehe , S. Liepelt , et al. 2010 Tree endurance on the Tibetan Plateau marks the world's highest known tree line of the Last Glacial Maximum. New Phytol. 185:332–342. 19761444 10.1111/j.1469-8137.2009.03007.x

[ece31840-bib-0031] Phillips, S. J. , R. P. Anderson , and R. E. Schapire . 2006 Maximum entropy modeling of species geographic distributions. Ecol. Modell. 190:231–259.

[ece31840-bib-0032] Qiu, Y. X. , C. X. Fu , and H. P. Comes . 2011 Plant molecular phylogeography in China and adjacent regions: tracing the genetic imprints of Quaternary climate and environmental change in the world's most diverse temperate flora. Mol. Phylogenet. Evol. 59:225–244. 21292014 10.1016/j.ympev.2011.01.012

[ece31840-bib-0033] Sandel, B. , L. Arge , B. Dalsgaard , R. G. Davies , K. J. Gaston , W. J. Sutherland , et al. 2011 The influence of late quaternary climate‐change velocity on species endemism. Science 334:660–664. 21979937 10.1126/science.1210173

[ece31840-bib-0034] Schaal, B. A. , D. A. Hayworth , K. M. Olsen , J. T. Rauscher , and W. A. Smith . 1998 Phylogeographic studies in plants: problems and prospects. Mol. Ecol. 7:465–474.

[ece31840-bib-0035] Singh, J. S. , and S. P. Singh . 1987 Forest vegetation of the Himalaya. Bot. Rev. 53:80–192.

[ece31840-bib-0036] Soltis, D. E. , A. B. Morris , J. S. McLachlan , P. S. Manos , and P. S. Soltis . 2006 Comparative phylogeography of unglaciated eastern North America. Mol. Ecol. 15:4261–4293. 17107465 10.1111/j.1365-294X.2006.03061.x

[ece31840-bib-0037] Song, Z. C. , and J. L. Liu . 1982 The Tertiary sporo‐pollen assemblages from Namling of Xizang Pp. 153–164 *in* Academia Sinica , ed. The comprehensive scientific expedition to the qinghai‐xizang plateau, Palaeontology of Xizang, vol. 5. Science Press, Beijing.

[ece31840-bib-0038] Sork, V. L. , and P. E. Smouse . 2006 Genetic analysis of landscape connectivity in tree populations. Landsc. Ecol. 21:821–836.

[ece31840-bib-0039] Taberlet, P. , L. Fumagalli , A. G. Wust Saucy , and J. F. Cosson . 1998 Comparative phylogeography and postglacial colonization routes in Europe. Mol. Ecol. 7:453–464. 9628000 10.1046/j.1365-294x.1998.00289.x

[ece31840-bib-0040] Tamura, K. , and M. Nei . 1993 Estimation of the number of nucleotide substitutions in the control region of mitochondrial DNA in humans and chimpanzees. Mol. Biol. Evol. 10:512–526. 8336541 10.1093/oxfordjournals.molbev.a040023

[ece31840-bib-0041] Tang, L. Y. , and C. M. Shen . 1996 Late Cenozoic vegetational history and climatic characteristics of the Qinghai‐Tibetan Plateau. Acta Micropalaeontol. Sin. 13:321–338.

[ece31840-bib-0042] Tischendorf, L. , and L. Fahrig . 2000 How should we measure landscape connectivity? Landsc. Ecol. 15:633–641.

[ece31840-bib-0043] Vandergast, A. G. , W. M. Perry , R. V. Lugo , and S. A. Hathaway . 2011 Genetic landscapes GIS toolbox: tools to map patterns of genetic divergence and diversity. Mol. Ecol. Resour. 11:158–161. 21429115 10.1111/j.1755-0998.2010.02904.x

[ece31840-bib-0044] Veloz, S. D. 2009 Spatially autocorrelated sampling falsely inflates measures of accuracy for presence‐only niche models. J. Biogeogr. 36:2290–2299.

[ece31840-bib-0045] Vignieri, S. N. 2005 Streams over mountains: influence of riparian connectivity on gene flow in the Pacific jumping mouse (*Zapus trinotatus*). Mol. Ecol. 14:1925–1937. 15910316 10.1111/j.1365-294X.2005.02568.x

[ece31840-bib-0046] Wang, I. J. , W. K. Savage , and H. S. Bradley . 2009 Landscape genetics and least‐cost path analysis reveal unexpected dispersal routes in the California tiger salamander (*Ambystoma californiense*). Mol. Ecol. 18:1365–1374. 19368644 10.1111/j.1365-294X.2009.04122.x

[ece31840-bib-0047] Wen, J. , J. Zhang , Z. Nie , Y. Zhong , and H. Sun . 2014 Evolutionary diversifications of plants on the Qinghai‐Tibetan Plateau. Front. Genet. 5:1–16. 24575120 10.3389/fgene.2014.00004 PMC3921583

[ece31840-bib-0048] Wu, Z. Y. 1988a Hengduan Mountain flora and her significance. J. Japan. Bot. 63:1–15.

[ece31840-bib-0049] Wu, Z. Y. 1988b Origin and evolution of the flora of Tibet Pp. 874–902 *in* Wu Z. Y., ed. Flora of Xizangica. Science Press, Beijing.

[ece31840-bib-0050] Xu, B. , Z. M. Li , and H. Sun . 2014 Plant diversity and floristic characters of the alpine subnival belt flora in the Hengduan Mountains, SW China. J. Syst. Evol. 52:271–279.

[ece31840-bib-0051] Yang, F. S. , Y. F. Li , X. Ding , and X. Q. Wang . 2008 Extensive population expansion of *Pedicularis longiflora* (Orobanchaceae) on the Qinghai‐Tibetan Plateau and its correlation with the Quaternary climate change. Mol. Ecol. 17:5135–5145. 19017264 10.1111/j.1365-294X.2008.03976.x

[ece31840-bib-0052] Yu, H. B. , and Y. L. Zhang . 2013 Advances in phylogeography of alpine plants in the Tibetan Plateau and adjacent regions. Acta Bot. Boreal‐Occident Sin. 33:1268–1278.

[ece31840-bib-0053] Yu, H. B. , Y. L. Zhang , S. C. Li , and W. Qi . 2014 Predicting the dispersal routes of alpine plant *Pedicularis longiflora* (Orobanchaceae) based on GIS and species distribution models. Chin. J. Appl. Ecol. 25:1669–1673. 25223022

[ece31840-bib-0054] Yue, L. L. , and H. Sun . 2014 Montane refugia isolation and plateau population expansion: phylogeography of Sino‐Himalayan endemic *Spenceria ramalana* (Rosaceae). J. Syst. Evol. 52:326–340.

[ece31840-bib-0055] Zeller, K. A. , K. McGarigal , and A. R. Whiteley . 2012 Estimating landscape resistance to movement: a review. Landsc. Ecol. 27:777–797.

