# Supplementary material for: Combining the least cost path method with population genetic data and species distribution models to identify landscape connectivity during the late Quaternary in Himalayan hemlock
Source: Ecol Evol. 2015 Nov 24;5(24):5781–91. doi: 10.1002/ece3.1840 (PMC4717335; doi:10.1002/ece3.1840)
Supplement: Supplementary file 1 — Table S1. Codes used for 19 bioclimatic variables. Table S2. Spearman's rank correlation coefficient (r) for the non‐eliminated bio‐climate predictors in the three periods. [file ECE3-5-5781-s001.docx]

Table S1 Codes used for 19 bioclimatic variables

| Code | Description | Code | Description |
| --- | --- | --- | --- |
| Bio1 | Mean annual temperature | Bio11 | Mean temperature of coldest quarter |
| Bio2 | Mean diurnal range (Mean of monthly (max temp - min temp)) | Bio12 | Annual precipitation |
| Bio3 | Isothermality (Bio2/Bio7) (*100) | Bio13 | Precipitation of wettest month |
| Bio4 | Temperature seasonality (standard deviation *100) | Bio14 | Precipitation of driest month |
| Bio5 | Max temperature of warmest month | Bio15 | Precipitation seasonality (Coefficient of Variation) |
| Bio6 | Min temperature of coldest month | Bio16 | Precipitation of wettest quarter |
| Bio7 | Temperature annual range (Bio5-Bio6) | Bio17 | Precipitation of driest quarter |
| Bio8 | Mean temperature of wettest quarter | Bio18 | Precipitation of warmest quarter |
| Bio9 | Mean temperature of driest quarter | Bio19 | Precipitation of coldest quarter |
| Bio10 | Mean temperature of warmest quarter |  |  |

Table S2. Spearman’s rank correlation coefficient (*r*) for the non-eliminated bio-climate predictors in the three periods

|  | BIO 1 | BIO 2 | BIO 3 | BIO 4 | BIO 12 | BIO 14 | BIO 15 | BIO 19 |
| --- | --- | --- | --- | --- | --- | --- | --- | --- |
| **Current** |  |  |  |  |  |  |  |  |
| BIO 1 |  |  |  |  |  |  |  |  |
| BIO 2 | -0.576 |  |  |  |  |  |  |  |
| BIO 3 | -0.037 | 0.398 |  |  |  |  |  |  |
| BIO 4 | -0.670 | 0.436 | -0.568 |  |  |  |  |  |
| BIO 12 | 0.673 | -0.606 | 0.165 | -0.720 |  |  |  |  |
| BIO 14 | 0.281 | -0.670 | -0.166 | -0.273 | 0.410 |  |  |  |
| BIO 15 | -0.038 | 0.560 | -0.025 | 0.323 | -0.237 | -0.631 |  |  |
| BIO 19 | 0.313 | -0.563 | -0.055 | -0.354 | 0.453 | 0.682 | -0.516 |  |
| **LGM** |  |  |  |  |  |  |  |  |
| BIO 1 |  |  |  |  |  |  |  |  |
| BIO 2 | -0.326 |  |  |  |  |  |  |  |
| BIO 3 | 0.066 | 0.611 |  |  |  |  |  |  |
| BIO 4 | -0.665 | 0.211 | -0.574 |  |  |  |  |  |
| BIO 12 | 0.595 | -0.602 | 0.030 | -0.685 |  |  |  |  |
| BIO 14 | 0.175 | -0.540 | -0.189 | -0.202 | 0.499 |  |  |  |
| BIO 15 | 0.015 | 0.387 | -0.085 | 0.340 | -0.352 | -0.605 |  |  |
| **LIG** |  |  |  |  |  |  |  |  |
| BIO 1 |  |  |  |  |  |  |  |  |
| BIO 2 | -0.470 |  |  |  |  |  |  |  |
| BIO 3 | 0.150 | 0.362 |  |  |  |  |  |  |
| BIO 12 | 0.452 | -0.263 | 0.108 |  |  |  |  |  |
| BIO 14 | -0.122 | -0.175 | -0.230 |  | 0.054 |  |  |  |
| BIO 15 | -0.632 | 0.578 | -0.378 |  | -0.461 | -0.079 |  |  |
| BIO 19 | -0.016 | -0.141 | -0.243 |  | 0.351 | 0.657 | -0.020 |  |

LGM, Last Glacial Maximum; LIG, Last Interglacial.
